# Assessment for Fuel Consumption and Exhaust Emissions of China's Vehicles: Future Trends and Policy Implications

**DOI:** 10.1100/2012/591343

**Published:** 2012-12-05

**Authors:** Yingying Wu, Peng Zhao, Hongwei Zhang, Yuan Wang, Guozhu Mao

**Affiliations:** School of Environmental Science and Engineering, Tianjin University, Tianjin 300072, China

## Abstract

In the recent years, China's auto industry develops rapidly, thus bringing a series of burdens to society and environment. This paper uses Logistic model to simulate the future trend of China's vehicle population and finds that China's auto industry would come into high speed development time during 2020–2050. Moreover, this paper predicts vehicles' fuel consumption and exhaust emissions (CO, HC, NO_*x*_, and PM) and quantificationally evaluates related industry policies. It can be concluded that (1) by 2020, China should develop at least 47 million medium/heavy hybrid cars to prevent the growth of vehicle fuel consumption; (2) China should take the more stringent vehicle emission standard V over 2017–2021 to hold back the growth of exhaust emissions; (3) developing new energy vehicles is the most effective measure to ease the pressure brought by auto industry.

## 1. Introduction

During the past decade, China's automotive industry has experienced a dramatically growth. Since 2009 China had become the largest vehicle producer and consumer. Auto industry has become a pillar industry in China's national economy and played an increasingly important role in economic development, employment promotion and domestic demand stimulation.

In recent years, the auto industry in developed countries has tended saturated state, with a slow growth (e.g., America) even a negative growth (e.g., Japan and Germany) in vehicle population. However, compared with developed countries, the per capita car ownership in China is very low and auto industry is still in a rapid increasing period. By the end of 2008, China's vehicle population was 51 million, just ranked 3rd after America and Japan (Table 1), and by August 2011, it has reached 93.5 million, second only to America (about 240 million) in the world. On a global scale, per capita car ownership is 0.15, while China's is 0.06, only 40% of the world average level. Calculated by the average level of the world, vehicle population in China should be 207 million, and that should reach 1.24 billion according to the American average level of 0.82 cars per capita. Obviously, it would bring severe challenges for China, including resource demand and exhaust emissions.

In fact, the crude oil consumption in China had reached 383.845 million tons in 2009, with average annual growth rate of 6.5% from 1990s, which is 4 times greater than the world growth rate (1.5%) over the same period. One of the major drivers of this increase in China's oil consumption is the rapid growth of the transportation sector in general and motor vehicles in particular [1]. About 60% of China's oil consumption in 2009 was for transportation [2]. Similarly, vehicle exhaust emissions grew from 17.24 million tons in 1995 to 40.555 million tons in 2009, with an annual average growth rate of 6.3% [3]. Thus, while auto industry expands rapidly, these environmental problems will become more stand out, seriously impeding the sustainable development of China.

Concerning the rapid development of the auto industry in China, scholars in the recent years have begun to keen to study a series of environmental issues associated with auto industry, analyzing the status, and predicting the future development. For example, Yan and Crookes [4] set two scenarios, best case scenario and baseline scenario, to assess the reduction potentials of energy demand and greenhouse gas (GHG) emissions in China's road transport sector till 2030. Hao et al. [5] established a bottom-up model to deliver the future trends of fuel consumption and life cycle GHG emissions by China's on-road trucks.

Meanwhile, the study in this field is also hot around the world. Dargay and Gately [6] analyzed and predicted the worldwide vehicle shock from 1960 to 2015, on the view of per capita income. Bastani et al. [7] predicted the uncertainty on US transport-related GHG emissions and fuel consumption out to 2050 and got the view that developing new energy vehicles is the necessary choice for auto industry, but negative influences brought about should not be ignored. Kloess and Müller [8] assess the energy consumption and GHG emissions on the passenger car fleet in Austria, from the aspect of policy, energy prices, and technological progress, and then concluded that material cuts and appropriate taxation on fuels and cars are positive measures for the development of auto industry. In addition, scholars also did studies on different fields, such as fuel types, energy efficiency, and industry policy, and discussed the fuel consumption and GHG emission in Malaysia [9], Europe [10, 11], China [12], and other countries [13].

However, the current literature focus little on quantification of specific implementation effects of auto industry policies. Additionally, the concentration on vehicle emissions is much more on GHG [4, 7–13] while it is little on the prediction of air pollutants (such as CO, HC, NO_*x*_, and PM). This paper aims to make up the defects in research, by predicting and evaluating the growth of auto industry in China during the next few decades, including vehicle population, fuel consumption, and vehicle exhaust emissions.

## 2. Prediction of Vehicle Population

### 2.1. Methodology

The change of vehicle population should be traced back to the combined actions of inner needs and external environmental restrictions, which could be properly described by Logistic equation. Logistic equation is a model to describe the trend of dependent variable over time. It can properly reflect the market expansion of new products [14] and shows the following characters: slow growth of the dependent variable at the initial stage, then experiencing a rapid increase stage, finally entering a market saturation stage.

As stated above, the total number of vehicles in China has been considerable. However, from the perspective of per capita car ownership, the auto industry in China is just at the primary phase, comparing with developed countries and even the world average level (Table 1). Thus, this paper assimilated China's auto industry to a market expansion process of new product and then forecasted the developing trend of China's vehicle population according to Logistic model.

Compared with other similar prediction models, for example, the Gompertz model [19, 20], the superiorities of Logistic model can be concluded to two aspects: few constraint conditions, and only one parameter estimation which the whole process involves. In fact, there have been some works in the literature applying Logistic model to the prediction of vehicle population in other countries. Button et al. [21] modeled vehicle ownership and use in five groups of low-income countries by quasilogistic model. Singh developed projections for future mobility in India, based on Logistic and Gompertz models, respectively [22].

The differential form of Logistic model can be expressed as [23]

(1) dFtdt=aFt(1−Ft),

where *F*_*t*_ = *N*_*t*_/*N*_*m*_,  *N*_*t*_ represents vehicle population at moment *t*, *N*_*m*_ is the maximum based on the market, and *a* is the instantaneous rate of increase, a constant. While (1 − *F*_*t*_) > 0, the vehicle population increases; while (1 − *F*_*t*_) < 0, it decreases; while (1 − *F*_*t*_) = 0, it remains stable.

Solving (1) by separating variables, we can see that

**Figure 1:**
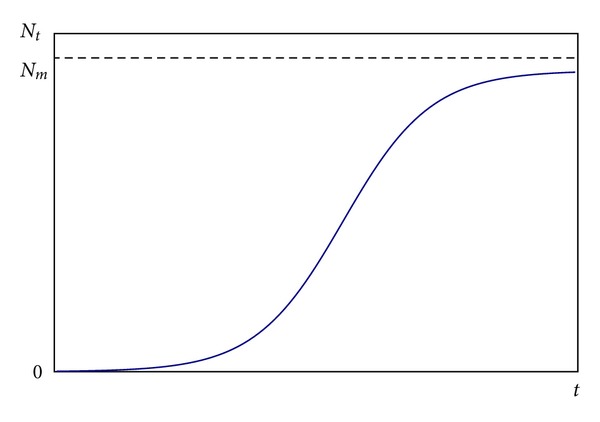
Logistic model curve.

(2) Ft=11+e−(b+at),

where *b* is a constant. Equation (2) is shown in Figure 1, which indicates that the Logistic model is an S-shaped growth curve. A feedback regulation exists among three variables (*F*_*t*_, (1 − *F*_*t*_), *a*), which makes vehicle population tend to the maximum *N*_*m*_.

Take the logarithm of (2):

(3) ln⁡Ft1−Ft=at+b.

It can be concluded from (3) that ln⁡*F*_*t*_/(1 − *F*_*t*_), with *t*, constitutes a linear relationship. Hence, estimating a maximum population value *N*_*m*_ based on the actual situation of our country, then figuring out ln⁡*F*_*t*_/(1 − *F*_*t*_), we can get the model parameters (*a* and *b*).

### 2.2. Model Calculation and Estimation Results

This paper consulted the per capita car ownership data (Figure 1) and population densities of developed countries to estimate *N*_*m*_. In 2008, Americans owned 0.82 cars per capita, while this value ranged from 0.54 to 0.69 in some other developed countries, such as Japan, Germany, Italy, Britain, France, and Spain. The auto industries had developed to a mature period in these countries above, even showing a negative growth since 2007 in the US, Japan, and Germany. Moreover, while the population density in the US (32 people per km^2^) was low, the value in Italy (196 people per km^2^) and France (113 people per km^2^) was closer to that in China (138 people per km^2^). Thus, this paper assumed that the per capita car ownership in China would be 0.6 when reaching the maximum. Suppose that Chinese population would keep around 1.4 billion in the next few decades, so *N*_*m*_ was calculated to be 840 million.

Calculate the model parameters (*a* and *b*) by (3) and show the result in (4). The original data were the statistic vehicle population in China from 1978 to 2008 (Table 2):

(4) ln⁡Ft1−Ft=0.1217t−6.5971.

**Figure 2:**
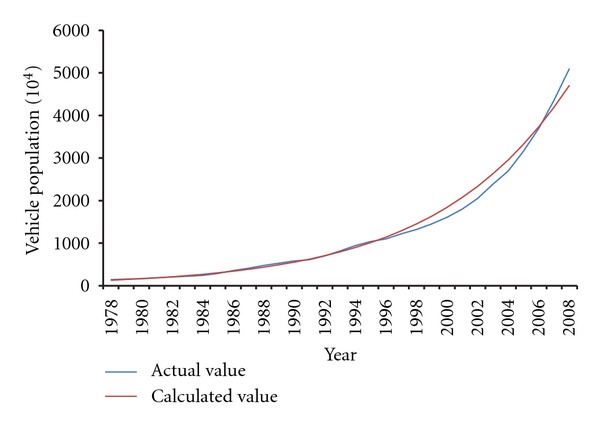
Comparison of calculated vehicle population and actual value.

The correlation coefficient *R* ^2^ of (4) was 0.9932, showing a good linear relationship. Draw the calculated curve by (4) and compare with the actual value in Figure 2. The fitting degree in Figure 2 was tested to be 98.76%, which could guarantee the validity of this model.

**Figure 3:**
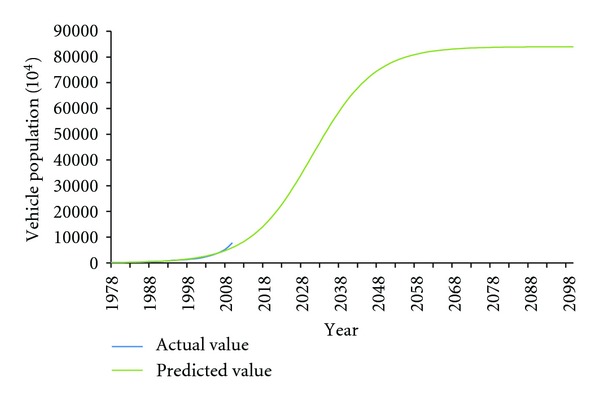
Prediction curve of future vehicle population in China.

The forecast outcome of future vehicle population in China was shown in Figure 3. The vehicle population in China would reach 171.02 million in 2020 and then enter a high speed growth period. In 2035, it would achieve 515.23 million, and at the same time the growth rate would begin to slow down. Around 2050, the vehicle population would have been 762.55 million, and then it would turn into a smooth growth period. Thus, the vehicle population in China should run through a quite rapid growth period inevitably over 2020–2050. Moreover, the enormous environmental pressure followed would bring China huge challenges, such as fuel consumption, exhaust emissions, and scrap car disposal.

## 3. Prediction of Fuel Consumption

According to the average vehicle fuel consumption per hundred kilometers and future vehicle population predicted above, this paper proposed a prediction of fuel consumption of future auto industry in China. The equation was as follows:

(5) Vt=10−5·vtSNt,

where *V*_*t*_ is the total vehicle fuel consumption of China in year *t*, 10^4^ m^3^; *v*_*t*_ is the average fuel consumption per hundred kilometers per car in year *t*, L/100 km; *S* is the annual average driving distance per car in China, km; *N*_*t*_ is the predictive value of vehicle population in year *t*, 10^4^.

In addition, *v*_*t*_ could be calculated on the basis of current statuses and planning objectives both in China and overseas (Table 3). The value of *S* was 2.42 × 10^4^ km, according to Table 3 and the total fuel consumption in 2010.

### 3.1. Scenario Prediction

Depending on different planning objectives for fuel consumption per hundred kilometers, it is proposed to set the following two scenarios.

**Scenario A**

In terms of average fuel consumption per hundred kilometers, Chinese auto industry develops under the current domestic set of development goals, which is described as the 1st line in Table 3.

**Scenario B**

The average fuel consumption of hundred kilometers in China will reach the world's most advanced level in 2025, which is equivalent to the EU fuel consumption level (the 3rd line in Table 3). That is to say, in 2018 the average level in China is assumed to achieve 5.46 L/100 km, the level of EU in 2015, and in 2025 it should achieve 2.94 L/100 km, equal to that of EU.

**Figure 4:**
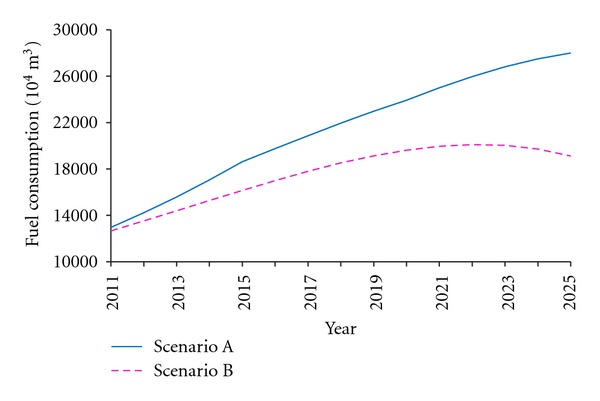
Forecast curve of China's vehicle fuel consumption from 2011 to 2025.

Calculate all the data in (5) and attain the forecast value of fuel consumption under the above two scenarios, shown in Figure 4.

In Scenario A, the fuel consumption in China will increase year by year in the next 15 years, with no trend to slow down. In 2022, the fuel consumption (239.07 million m^3^) will be twice than that in 2011 (121.31 million m^3^). In Scenario B, the fuel consumption will achieve the largest in 2022 (185.10 million m^3^) and then begin to decline. The accumulative fuel consumptions from 2011 to 2025 will have been 2.3 billion tons and 1.9 billion tons, respectively. However, the Chinese oil reserve was only 2.95 billion tons in 2009. According to the developing trend in Scenario A, the oil reserve in China would be depleted out before 2030 without relying on oil import.

### 3.2. Fuel Consumption Policy Evaluation

In July 2012, China has officially released Energy Saving and New Energy Vehicles Industry Development Planning (2012–2020) (hereinafter referred to as the “*Planning*"), which detailed goals of energy saving and new energy vehicles in China. In* Planning*, there are two stage goals on the market share of new energy vehicles: (1) up to 2015, the number of pure electric vehicles (PEVs) and plug-in hybrid electric vehicles (PHEVs) should outnumber 0.5 million, and medium/heavy hybrid cars should reach one million; (2) up till 2020, the number of PEVs and PHEVs should reach 5 million, and medium/heavy hybrid cars should popularize on a large scale.

**Figure 5:**
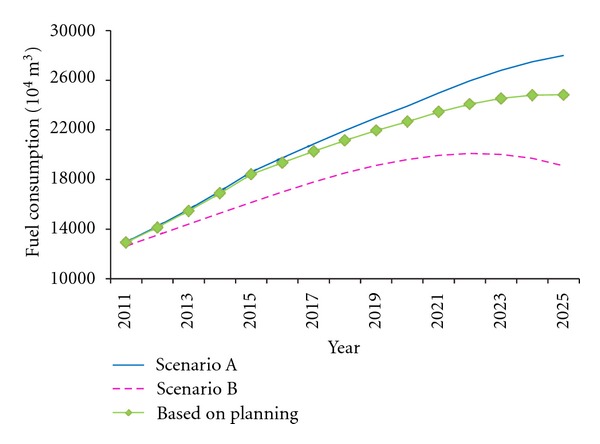
Forecast curve of China's vehicle fuel consumption under the implementation of *Planning*.

To assess the effect of *Planning*, this paper contrasts it with the two scenarios above, shown in Figure 5. From Figure 5, we can see that according to *Planning*, the fuel consumption in China would still keep in a rising trend, but since 2015 the growth will slow down significantly. The fuel consumption would be 1.18% lower than that in Scenario A by 2015, and 9.72% lower by 2020. Thus, we conclude that developing new energy vehicles is the absolutely necessary to reduce the fuel consumption in China. However, to achieve the world's most advanced level, the developing track in Scenario B, China would continue to reduce to 86% of the total fuel consumption by 2015, and 89% by 2020, on the base of *Planning*. That is, by 2020, under the premise of completing the target of 5 million PEVs and PHEVs, medium/heavy hybrid cars should be developed to 47 million at least, which is equivalent to 60% of current domestic vehicle population. Thus, China faces difficult challenges to reduce the vehicle fuel consumption.

## 4. Prediction of Exhaust Emissions

Since 2000, when Motor Vehicles Emission Standard I implemented, China has implemented 4 emission standards, as shown in Table 4. According to* China Vehicle Emission Control Annual Report (2010) *[3], the reduction effects of stringent implement of motor vehicle emission standards emission is very significant. In 2009, vehicles produced before Standard I, which just accounted for 17.1% of the total, discharged four main pollutants (CO, HC, NO_*x*_, and PM) in excess of 50%, while vehicles produced after Standard III, which made up 25.4% of the total, only discharged 4% of the four pollutants.

### 4.1. Scenario Prediction

To predict and assess changes of four major pollutants (CO, HC, NO_*x*_, and PM) in vehicle emissions, the paper intends to figure out the annual vehicle exhaust emissions by:

(6) Mt,k=∑imt,kNi,t,

where *M*_*t*,*k*_ is the emissions of pollutant *k* in year *t*, 10^4^ *t*; *m*_*i*,*k*_ is the annual emissions of pollutant *k* per car performing Standard *i* (Table 5), *t*; *N*_*i*,*t*_ is the predictive value of vehicle population performing Standard *i* in year *t*, 10^4^; *i* presents Standard I before, Standard I, Standard II, Standard III, and Standard IV; *k* presents the four main exhaust pollutants, that is, CO, HC, NO_*x*_, and PM.

*N*_*i*,*k*_  is calculated by the relation of vehicles scrapped and vehicle sales per year. Accounting for the different vehicle life cycles, it is proposed to set the following two scenarios.

**Scenario A**

The vehicle life cycle is 15 years, the maximum prescribed years in* Motor Vehicle Rejection Standard (1997)* [24]. That is, vehicles before Standard I would be fully scrapped till 2014, by the same token, vehicles performing Standards I, II, and III would be fully scrapped, respectively till 2018, 2021, and 2027, while new vehicles would perform Standard IV.

**Scenario B**

The vehicle life cycle is 11 years, which is calculated according to *Motor Vehicle Rejection Standard (1997)* [24] and the portion of different vehicle types. Thus, vehicles before Standard I would be fully scrapped till 2010, by the same token, vehicles performing Standards I, II, and III would be fully scrapped, respectively till 2014, 2017, and 2023, while new vehicles before 2013 would perform Standard III, then would perform Standard IV.

**Figure 6:**
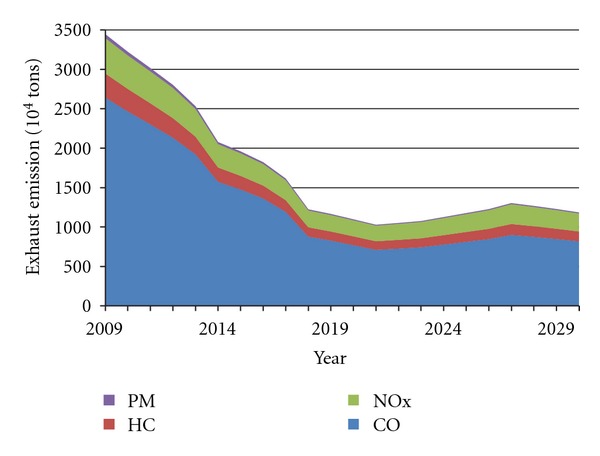
Forecast curve of China's exhaust emissions in Scenario A.

**Figure 7:**
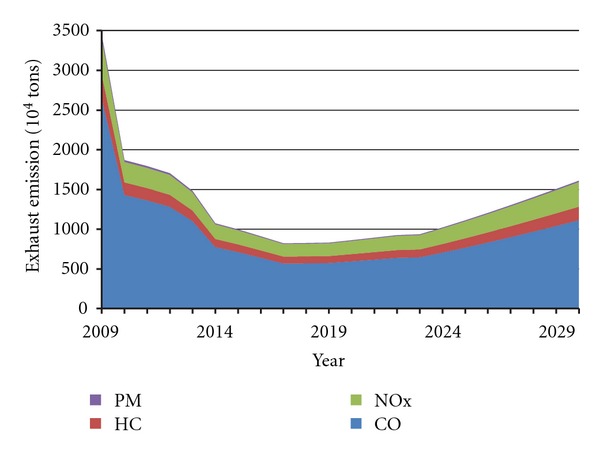
Forecast curve of China's exhaust emissions in Scenario B.

The prediction curves of China's four major pollutants in vehicle emissions under two scenarios are shown in Figures 6 and 7. It can be found that changes of four pollutants are roughly consistent under the two settings and show a trend that first decreases and then grows. At the lowest point, total emissions, respectively, amount to 10.28 million tons in 2021, and 8.22 million tons in 2017.

It can be concluded that prior to these two time points (2021 in Scenario A, and 2017 in Scenario B), the current implementation of emission standards would continue to play a role in the next 5–10 years, and then the increase in vehicle population would surpass the constraint function of Standard IV, resulting in the regrowth of pollutant emissions. Thus, to ensure the exhaust emissions growth, China should take the more stringent emission standard V at least in 2017–2021.

### 4.2. Exhaust Emission Policy Evaluation

**Figure 8:**
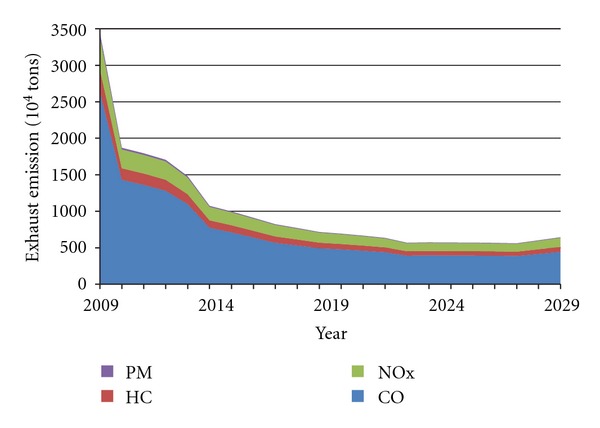
Forecast curve of China's exhaust emissions under the implementation of Standard V in Scenario A.

**Figure 9:**
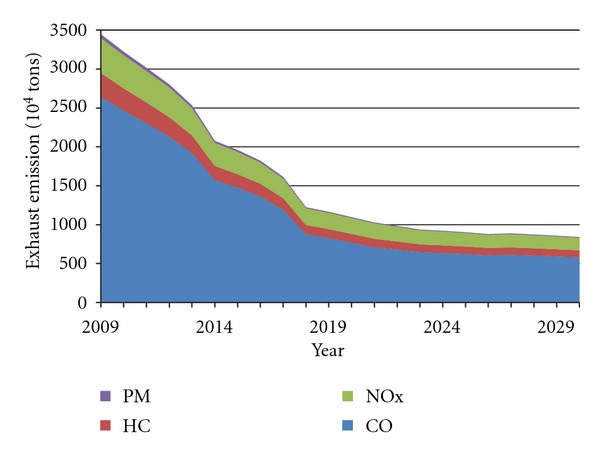
Forecast curve of China's exhaust emissions under the implementation of Standard V in Scenario B.

Further analysing the two scenarios and evaluating the effectiveness of emission standard V, we make two assumptions: firstly, Standard V was 60% lower on the basis of Standard IV, referring the reduction effects of Standard I to Standard IV; secondly, Standard V would be implemented after the time points of lowest emissions (2021 in Scenario A, and 2017 in Scenario B). The prediction curves are shown in Figures 8 and 9. It can be seen that, due to the timely implementation of Standard V, China's vehicle pollutant emissions continue to decline, but the rate is slowing down. Under Scenario B, emissions of the four pollutants restart the upward trend after 2028. However, Table 5 shows that the annual emission of four pollutants per car performing Standard V is only 0.016*t*, about 1/100 of that of vehicles before Standard I. If only implementing emission standard VI, rather than taking other measures, it would achieve little success, comparing the rapid growth of vehicle population. Therefore, to further control vehicle exhaust emissions, China should actively develop new energy vehicles, on the basis of continuing to strengthen phased emission standards.

## 5. Conclusions

In this paper, we developed a prediction of vehicle population in China in the next few decades, based on Logistic model, by using a statistical data set over the period of 1978–2008. Given the per capita car ownership and population density of developed countries, we assumed that per capita would own 0.6 cars when entering the saturation state. We predicted that the total number of China's vehicles would be approximately 15 times higher in 2050 than in 2008, increasing to more than 750 million vehicles (Figure 3). It also can be concluded that China's auto industry would run through a high speed development period during 2020–2050, which would inevitably aggravate China's environmental burden.

Moreover, fuel consumption prediction over 2011 to 2025, based on the average vehicle fuel consumption per hundred kilometers, shows that under the current scenario, China's fuel consumption for vehicles would grow without trend to slow down, reaching 239.07 million m^3^ in 2022 (Figure 4), which is the twice in 2011. However, under the world's most advanced scenario, it would peak in 2022 and then begin to decline. Considering the tension of fuel supply, the current development goals for China auto industry could not be suited to demand. Further, we evaluated *Planning*, China's latest policy for vehicle fuel consumption, and found that the growth trend of fuel consumption in China would meet a marked slowness since 2015 (Figure 5), on the basis of current development goals. Nevertheless, to achieve the world's most advanced level, China should develop medium/heavy hybrid cars to 47 million at least by 2020, under the existing premise of completing the target of 5 million PEVs and PHEVs.

Furthermore, allowing for different vehicle life cycles (the maximum and the average), exhaust emissions prediction for China's vehicles up to 2029 was developed in this paper, on the basis of vehicle emission standards for different phrases. We projected that the four pollutants (CO, HC, NO_*x*_, and PM) would display roughly consistent changing trends under the two settings and reach the lowest points, respectively, in 2021 and in 2017 (Figures 6 and 7). Thus, in order to hold back the exhaust emission growth, China should continue to implement the more stringent emission standard V at least in 2017–2021. However, further study on the effect of emission standards showed that the Standard VI would achieve little, when it meets the rapid growth of vehicle population (Figures 8 and 9). 

Finally, our results suggest that the future strong growth in China's vehicle population will bring huge burdens on fuel demand and exhaust emissions. China should actively develop new energy vehicles, which is the most effective measure to ease the pressure on fuel demand and exhaust emissions brought by auto industry.

## Figures and Tables

**Table 1:** Worldwide vehicle population and per capita car ownership in 2008.

Country	Vehicle population/10^4^	Per capita cars
USA	25024	0.82
Japan	7553	0.59
China	5100	0.04
Germany	4400	0.54
Italy	4089	0.69
Russia	3826	0.27
France	3721	0.60
Britain	3562	0.58
Spain	2761	0.60
Brazil	2748	0.14
Mexico	2531	0.24
Canada	2052	0.62
Poland	1909	0.50
India	1851	0.02
Korea	1679	0.35
Australia	1468	0.67
Turkey	1019	0.15
Thailand	977	0.15
The Netherlands	891	0.52
Argentina	846	0.21
Indonesia	825	0.04
South Africa	749	0.15
Belgium	586	0.53
Sweden	480	0.53
Austria	468	0.59
Switzerland	436	0.62
Others	15752	0.07

Total	97306	0.15

Data resource: National Bureau of Statistics of China, Japan Automobile Manufacturers Association, German Automobile Industry Association, Ward's Auto, and ANFIA.

**Table 2:** Vehicle population from 1978 to 2008 in China.

Year	1978	1979	1980	1981	1982	1983	1984	1985
Population	142.92	156.57	168.10	187.30	205.32	222.71	243.37	288.71

Year	1986	1987	1988	1989	1990	1991	1992	1993
Population	357.45	412.29	477.64	527.47	583.59	611.41	701.47	817.58

Year	1994	1995	1996	1997	1998	1999	2000	2001
Population	941.95	1040.00	1100.08	1219.09	1319.30	1452.94	1608.91	1802.04

Year	2002	2003	2004	2005	2006	2007	2008	
Population	2053.17	2382.93	2693.71	3159.66	3697.35	4358.36	5099.61	

Unit: 10^4^.

**Table 3:** Statuses and planning targets of domestic and foreign vehicle average fuel consumption per hundred kilometers per car.

	Current	2015	2020	2025
China	8.25^1^	7.5^2^	5.78^3^	—
Japan	6.13 [15]	—	4.93 [15]	—
EU	6.13 [16]	5.46 [15]	3.99 [15]	2.94 [16]
USA	7.50 [15]	—	—	4.31 [15]

Unit: L/100 km.

Tips: ^1^8.25 = (1 + 0.1) × 7.50, according to the report that current fuel consumption in China was 10% higher than that of USA (available on http://www.find800.cn/news/bus/ye/110930/12778.html).

^2^7.5 = 7 × 0.75 + 9 × 0.25, according to [17, 18] and weights for passenger car and commercial car in China.

^3^5.78 = 5 × 0.75 + 8.1 × 0.25, the same as Tip 2.

**Table 4:** Implementation schedule of various Motor Vehicles Emission Standard in China.

Year	2000–2003	2004–2006	2007–2010	2011	2012	2013
Emission standard	I	II	III	IV in batches		

**Table 5:** Annual emissions of 4 pollutants for various standards.

	I before	I	II	III	IV^1^
CO	1.4648	0.6421	0.2107	0.0715	0.0286
HC	0.1809	0.0662	0.0221	0.0110	0.0044
NO_*x*_	0.2477	0.0977	0.0400	0.0200	0.0080
PM	0.0296	0.0099	0.0036	0.0010	0.0004

Total	1.9230	0.8159	0.2766	0.1035	0.0414

Unit: tons/car.

Data resource: *China  Vehicle  Emission  Control  Annual  Report (2010)* [3].

Tip: ^1^According to news report, the data for Standard IV was 60% lower than Standard III (Available on http://auto.qq.com/a/20050530/000043.htm).
